# Improvised vacuum assisted closure dressing for enterocutenous fistula, a case report

**DOI:** 10.1016/j.ijscr.2020.11.049

**Published:** 2020-11-23

**Authors:** Masawa K. Nyamuryekunge, Biswalo Yango, Ally Mwanga, Athar Ali

**Affiliations:** a Surgery Department, The Aga Khan Hospital, Tanzania; b The Aga Khan University, Medical College, Dar es salaam Campus, Tanzania; c Department of Surgery, The Aga Khan Hospital, P.O. BOX 2289, Dar es Salaam, Tanzania

**Keywords:** Negative pressure wound therapy, Postoperative enterocutaneous fistula, Africa, Case report

## Abstract

**Introduction:**

Management of enterocutaneous fistula is challenging with high morbidities and mortalities despite the recent advances in surgical technique. The bad outcomes are a result of associated metabolic complications. Vacuum-assisted closure dressing for the management of enterocutaneous fistula is a relatively new technique with benefit as a bridge to definitive surgery or definitive management in achieving spontaneous closure at a shorter time. In the current report, we share our experience of improvising vacuum-assisted closure dressing for managing postoperative enterocutaneous fistula and achieving spontaneous closure

**Presentation of case:**

We describe a case of a 56-year-old male from Tanzanian with a postoperative discharge of intestinal contents from the wound. He was diagnosed to have a proximal enterocutaneous fistula. After sepsis control and achieving hemodynamic stability, the enterocutaneous fistula was managed with parenteral nutrition, proton pump inhibitors, anti-cathartics, and somatostatin analogs. Endoscopic therapies and fibrin sealants are other described nonoperative interventions for enterocutaneous fistula. The unavailability of these modalities limited us. Vacuum-assisted closure dressing was improvised using gauze pieces, feeding tube, and Op-site dressings at a pressure of −30 mmHg. We achieved spontaneous closure of the proximal enterocutaneous fistula in 32 days.

**Discussion:**

The time to closure was within the range of 12–90 described for conventional vacuum assisted closure dressing, and there were no complications. Close monitoring of improvised VAC dressings is required as the risks are unknown; however, given the known complications of conventional VAC dressing, a risk of hemorrhage and creation of entero-atmospheric fistula exists.

**Conclusion:**

Improvised VAC dressing for ECF is potentially an acceptable option with promising outcomes in low-resource settings.

## Highlights

• Enterocutaneous fistula is a postoperative complication is 75–80% of the cases that results in metabolic complications.
• Management of this complication is difficult, necessitating delayed surgery with associated high morbidity and significant mortality.
• Vacuum-assisted closure (VAC) dressing has been shown to decrease the healing time of chronic wounds and achieves a 64% spontaneous enterocutaneous fistula closure rate.
• We improvised VAC dressing using simple materials for proximal enterocutaneous fistula.
• Spontaneous closure was achieved on day 32.

## 1 Introduction

Vacuum-assisted closure (VAC) dressing for managing enterocutaneous fistula (ECF) has some benefit of achieving spontaneous ECF closure [1]. The management ECF follows sepsis control, hemodynamic stabilization, nutritional support, and wound care [2]. The prognosis is determined by the etiology, involved intestinal segment, and quantity of the effluent [3,4].

Spontaneous closure rates are low, and a chronic catabolic state causes 11% of the subsequent mortalities due to protein-energy malnutrition, hemodynamic, acid-base and electrolyte imbalances [5,6]. Delayed surgery is thus frequently offered for fistula excision, and in that time, patients are optimized, and a favorable peritoneal cavity is encountered [7,8]. The delay does not always achieve the intended goals as ongoing losses further diminish the patient's nutritional reserve and aggravate the metabolic derangements causing an attendant immune-compromised state and mortality [5]. Surgery is still associated with 90% morbidities and 20% mortality [[9], [10], [11], [12]]. Achieving early fistula closure without the need for surgery is desirable.

Described endoscopic therapies for ECF are deploying stents for exclusion, clipping, and fistula plugging of the internal opening. Accessing the small bowel endoscopically is challenging, and deployed stents may migrate. Clipping has 42.9% and plugging 80% closure rate [13]. Fibrin sealants require multiple sessions with an 86% closure rate [14].

VAC dressing reduces inflammatory debris and edema. Micro and macro deformation promotes angiogenesis, granulation tissue, wound contraction, decreased healing time by 78%, spontaneous closure rate of 64.6% in 58 days [1,15,16]. A significant complication is a new fistula formation (4%). Success is determined by the amount of effluent and length of fistula; when the mucosa is visible, spontaneous closure is not achieved [1]. In the current study from a private teaching hospital in Dar es salaam, Tanzania, we describe the first published report of improvised VAC dressing for postoperative ECF. The other non-surgical interventions were unavailable. Improvised VAC dressing can potentially be utilized in low-resource settings with good outcomes. This paper has been reported in line with the SCARE criteria [17].

## 2 Presentation of case

Our patient is a 56-year-old Tanzanian married man with three children. Familial diseases and drug allergies were not reported nor comorbidities or cigarettes, medications and alcohol use. He had a past surgical history of explorative laparotomy for multiple distal ileal and ascending colonic perforated necrotic patches in 2018. The etiology was undetermined.

In July 2020, he had colicky abdominal pain, worsening over five months. The general examination was unrevealing. Per the abdomen, apart from the prior surgical scar, he had no features of intestinal obstruction or peritonitis. With a clinical impression of chronic intestinal obstruction, with differentials of adhesions, CT scan abdomen with oral contrast showed a jejunal-ileal segment stricture (Appendix 1). He underwent adhesiolysis, resection, and anastomosis. Histology revealed inflammatory changes.

He was re-admitted 14 days later with intraabdominal sepsis. Localized purulent contamination caused by a pinpoint perforation at the ileum was found. An inadvertent unrecognized enterotomy was suspected as the cause. Within 72 h, he developed peritonitis again and was found to have a small perforated area in the transverse colon; end ileostomy was fashioned (Appendix 2).

### 2.1 Clinical features

Postoperatively he was admitted in ICU with a qSOFA score of 1, corresponding to a 7% risk of poor outcome and a SAPS II score of 15, corresponding to 2.0% mortality risk. Intravenous (IV) meropenem 1000 mg thrice daily for ten days, and intravenous fluids (IVF) titrated to maintain end-organ perfusion were started. He did not sustain organ failure.

He developed purulent discharge on the surgical wound. The septic parameters were normalizing, and no features of peritonitis. An impression of deep surgical site infection (SSI) was reached, with a differential of organ space SSI. Staples were released, finding a purulent collection with an intact rectus fascia. A sterile absorbent dressing was done, which soaked bilious fluid copiously on oral feeding and upright posture. Two areas on the wound floor, with a partial loosening of fascial sutures, were oozing bilious effluent (Appendix 3). Four dressings changes per day were required to keep the wound dry. A clinical impression of proximal, possibly moderate to high output ECF fistula was made.

### 2.2 Diagnostic assessment

CT-abdomen with oral gastrograffin was done to evaluate for generalized peritoneal contamination. Free contrast was not seen in the peritoneal cavity; however, some air bubbles could be seen tracking to the midline wound without distal intestinal obstruction (Appendix 4). Clinically and radiologically, this was a proximal ECF given the bilious contents, output between 200 to 500cc per day given dressing changes required. Furthermore, this was possibly a short fistulous tract, as tracking of contrast was not demonstrated. By four days in ICU, he had visible evidence of weight loss compared to when he presented initially in July, and he had serum albumin of 17.0 g/L.

By day 9, he had a qSOFA of 0, 3% risk of poor outcome, is unlikely to have sepsis. He was transferred from the ICU.

### 2.3 Therapeutic information

To control fistula effluent and prevent the accompanying metabolic derangements, total parenteral nutrition (TPN) was initiated progressively to a target of 31 K/Cal and protein allowance of 2.5 g per day. Other measures included NPO, loperamide 2 mg per oral daily, IV octreotide 100mcg thrice daily, and IV rabeprazole 20 mg daily. IVF 3lts per day was given to maintain euvolemia. Hypophosphatemia of 0.46 mmol/L, hypokalemia of 3.28 mmol/L, and hyponatremia of 128.75 mmol/L were corrected.

Apart from VAC dressing, endoscopic interventions for ECF and fibrin sealants were considered. These modalities are unavailable in our setting. Conventional VAC dressing is also not available in our setting; We improvised the VAC dressing. Improvisation proceeded by placing two layers of wet gauze pieces on the wound surface. With extra holes, a feeding tube wrapped around a sterile gauze piece was placed on top of the initial gauze layer. Another single layer of gauze piece was placed on top of the wrapped feeding tube. A water-resistant transparent sterile covering, OpSite, was then applied. The feeding tube was connected to a negative pressure of −30 mmHg (Appendix 5).

The tube was not placed directly over the wound to avoid causing hemorrhage and entero-atmospheric fistula. Gauze pieces were used to achieve equal distribution of negative pressure on the wound surface.

Close monitoring for evidence of negative pressure on the tubbing, hemorrhage, pain, and development of entero-atmospheric fistula was done, no complications occurred. The dressing was done by surgical interns and residents, requiring two people and 30 min for completion. It took medical interns unfamiliar with this dressing four assisted sessions to dressing independently.

### 2.4 Follow up and outcomes

Progressive granulation of the midline wound, clearance of debris, and wound contraction was noted (Appendix 5). On day 32, oral feeding was initiated and by day 40, he was on regular kitchen feeds meeting caloric and protein requirements without effluent. He reported weight gain subjectively, and biochemically his serum albumin was 31.50 g/L. He underwent secondary closure of midline wound and was discharged the following day. The follow-up management in outpatient was aimed at nutritional management and assessment for ileostomy related complications. This is the fourth-week post-discharge, with no evidence of a recurrence of ECF.

## 3 Discussion

In evaluating the jejunal-ileal structuring mass, enteroscopy would have enabled us to obtain a biopsy and rule out small bowel malignancy, obviating the initial surgery. The modality is unavailable in our setting [18]. ECF management followed the described phases with sepsis management, maintain hemodynamic stability, correcting metabolic derangements, and attention to the wound [2]. Further evaluation of the ECF anatomy could have been delineated with contrast meal and follow-through; however, the facility was unavailable during this period. To also assess for intraperitoneal collections, the CT abdomen was deemed a suitable option.

In this case, time to closure was 32 days, similar to a median of 58 days (12–90 days) reported in a systematic review by Misky et al. [1]. VAC dressings' mechanism of action depends on the dressing-wound interface to achieve adequate distribution of mechanical force essential in stimulating the correct amount of macro and micro deformation [15,19]. The specifications of mechanical force for efficient healing is still an ongoing research area; what is apparent is the potential to improve outcomes [1].

This study adds another possible facet to VAC dressings' specifications, showing that there might be a wide range of suitable mechanical specifications. Apart from fistula closure, there was a visible progression through wound healing stages, further outlining VAC dressings impact wound healing [15]. We had prior experience with improvised VAC dressing that has not yet been published; recently, we used it to manage a pancreatico-cutaneous fistula and achieving spontaneous closure. With growing cautious optimism, we hope to assess this method further.

Advantages include the use of low-cost materials and a short learning curve. Despite the success, in this case, caution is advised as several other factors govern ECF prognosis. The fistula length, diameter, and presence of distal obstruction determine prognosis. Spontaneous ECF requires treatment of the underlying condition. The risks of improvised VAC dressings are unknown; however, given the known complications of conventional VAC dressing, a risk of hemorrhage and the creation of entero-atmospheric fistula exists. Improvised VAC dressing for ECF is potentially an acceptable option with promising outcomes in low-resource settings.

## 4 Patient perspectives

It was a relief to be able to feed orally finally; it had been a challenging course. There was doubt, sadness, fear, and hopelessness that maybe the hole in the intestines would never close. It is a joy to be able to go home. God bless everyone involved for the hard work and kind care.

## Conflicts of interest

None.

## Funding

None.

## Ethical approval

Case study is exempt from ethical approval in my institution.

## Consent

Written informed consent was obtained from the patient for publication of this case report and accompanying images. A copy of the written consent is available for the review by the editor-in-chief of this journal on request.

## Author contribution

MASAWA KLINT- Study conceptualization, Data Collection, Preparation of Manuscript.

BISWALO YANGO- Data collection, Preparation of Manuscript.

ALLY MWANGA- Manuscript preparation, review, and Proof reading.

ALI ATHAR- Manuscript preparation, review, and Proof reading.

## Registration of research studies

1. Name of the registry: RESEARCH REGISTRY
2. Unique identifying number or registration ID: researchregistry6123
3. Hyperlink to your specific registration (must be publicly accessible and will be checked): www.researchregistry.com/browse-the-registry#home/?view_2_search=researchregistry6123&view_2_page=1

## Guarantor

Dr. Athar Ali.

## Provenance and peer review

Not commissioned, externally peer-reviewed.
