# Supplementary material for: Improvised vacuum assisted closure dressing for enterocutenous fistula, a case report
Source: Int J Surg Case Rep. 2020 Nov 23;77:610–3. doi: 10.1016/j.ijscr.2020.11.049 (PMC7708767; doi:10.1016/j.ijscr.2020.11.049)
Supplement: Supplementary file 1 [file mmc1.docx]

**APPENDICES**


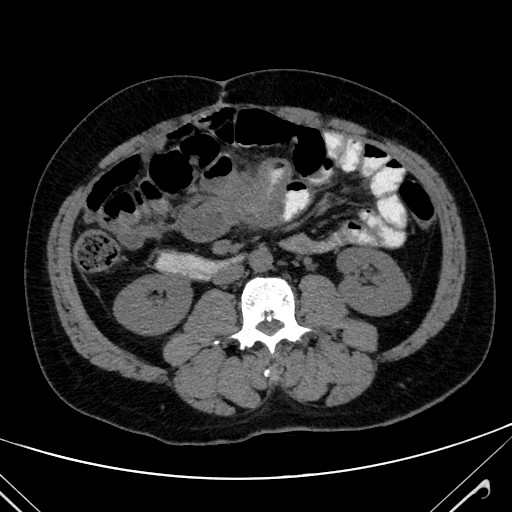


Appendix 1: CT of Jejunal-ileal Stricture

CT Abdomen with oral contrast acquired on the 1^st^ August 2020. Cross-sectional image showing area of thickening and fat stranding on the distal jejunum.


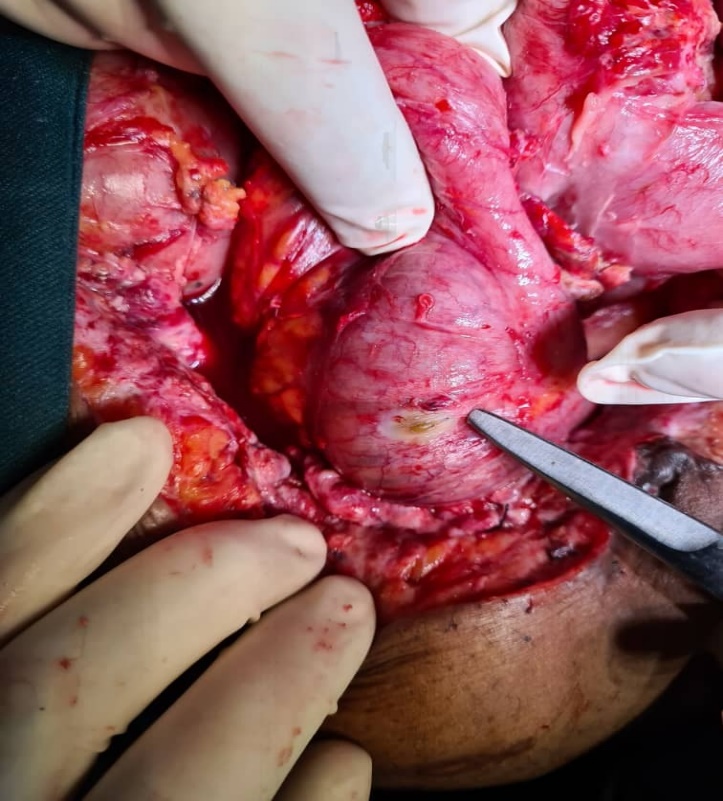


Appendix 2: Suspected inadvertent Enterotomy

Intraoperative image showing perforation on transverse colon, suspected to be an inadvertent unrecognized enterotomy.


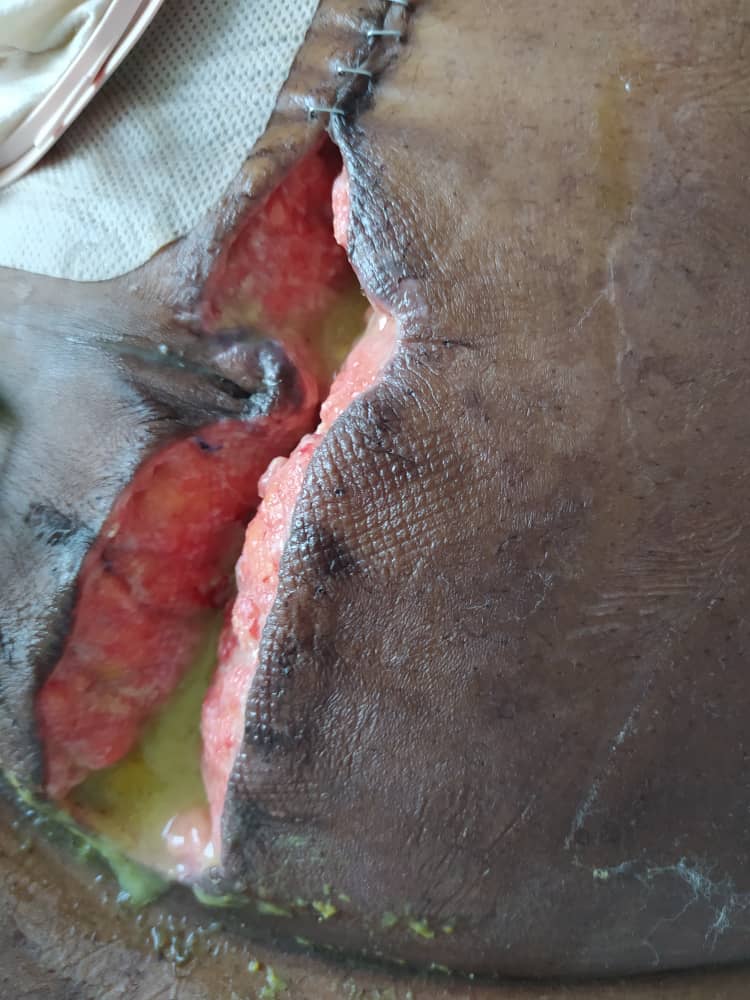


Appendix 2: macroscopic evidence of ECF

Image of released laparotomy wound. Collection of bilious contents on floor of the wound


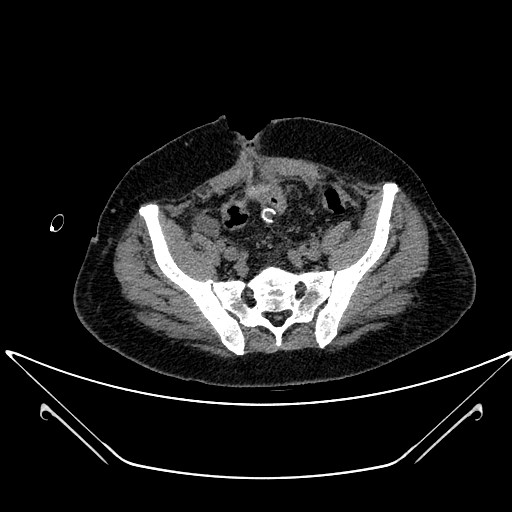


Appendix 3: CT ECF image

CT Abdomen with oral contrast. Cross-sectional image showing air bubbles tracking to wound surface. No collections or free contrast in peritoneal cavity seen.


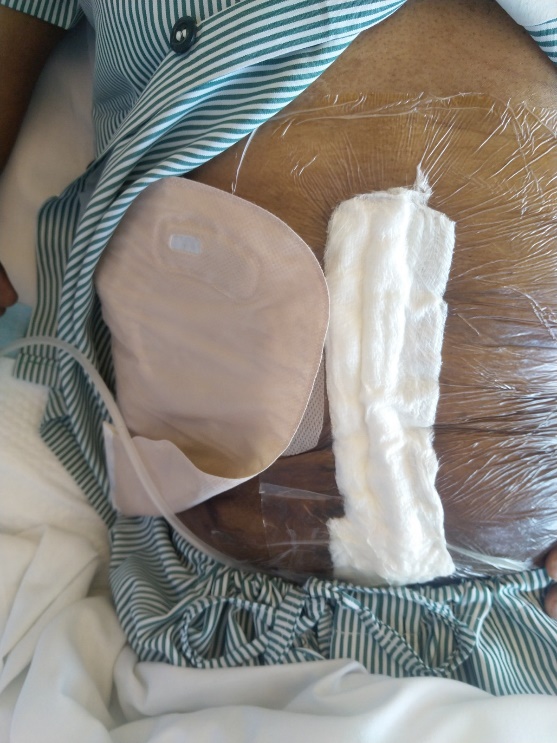


Appendix 4: Improvised VAC Dressing

Image showing Improvised VAC dressing. Gauze pieces seen through OpSite covering with evidence of vacuum. Tubbing can be seen from dressings.


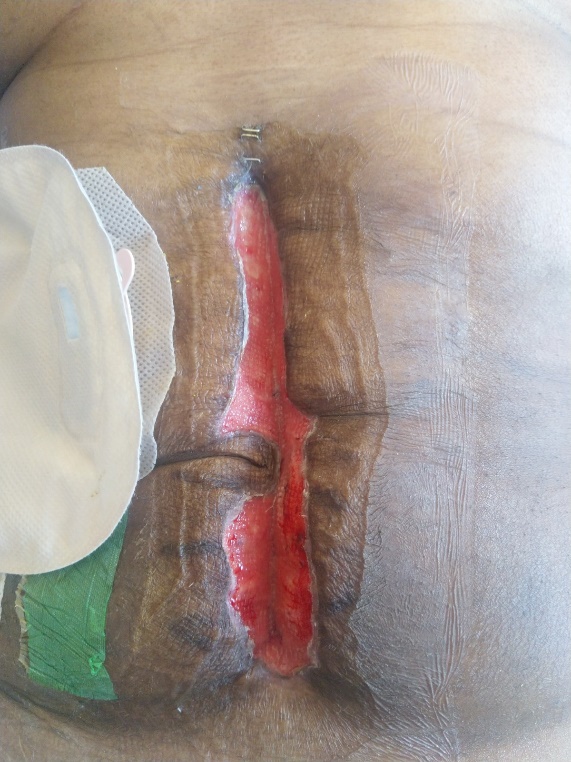


Appendix 5: Healing Laparotomy Wound.

Image of wound with evidence of granulation, wound contraction and no billious contents in wound floor.
